# In Vivo and In Vitro Effects of Tracheloside on Colorectal Cancer Cell Proliferation and Metastasis

**DOI:** 10.3390/antiox10040513

**Published:** 2021-03-25

**Authors:** Min-Kyoung Shin, Yong-Deok Jeon, Seung-Heon Hong, Sa-Haeng Kang, Ji-Ye Kee, Jong-Sik Jin

**Affiliations:** 1 Department of Oriental Medicine Resources, Jeonbuk National University, 79 Gobong-ro, Iksan 54596, Korea; smksin0807@gmail.com (M.-K.S.); kangsh@jbnu.ac.kr (S.-H.K.); 2 Institute of Natural Medicine, University of Toyama, 2630 Sugitani, Toyama 930-0194, Japan; 3 Department of Oriental Medicine Resources, Woosuk University, 443 Samnye-ro, Samnye-eup, Wanju-Gun 55338, Korea; dugicom@nate.com; 4 Department of Oriental Pharmacy, College of Pharmacy, Wonkwang-Oriental Medicines Research Institute, Wonkwang University, Iksan 54538, Korea; jooklim@wku.ac.kr; 5 Advanced Institute of Environment and Bioscience, Jeonbuk National University, 79 Gobong-ro, Iksan 54596, Korea

**Keywords:** phenolic compound, tracheloside, anti-oxidant, apoptosis, EMT, metastasis, colorectal cancer

## Abstract

Recent research suggests a relationship between cancer progression and oxidative mechanisms. Among the phenolic compounds such as tracheloside (TCS) are a major bioactive compound that can combat oxidant stress-related chronic diseases and that also displays anti-tumor activity. Although TCS can inhibit mammalian carcinoma, its effects on colorectal cancer (CRC) have not been clarified. The purpose of this study was to investigate the effects of TCS on the proliferation of CRC cells, the metastasis of CT26 cells, and the molecular mechanisms related to TCS in vitro and in vivo. A cell viability assay showed that TCS inhibited the proliferation of CRC cells. TCS-treated CT26 cells were associated with the upregulation of p16 as well as the downregulation of cyclin D1 and CDK4 in cell cycle arrest. In addition, TCS induced apoptosis of CT26 cells through mitochondria-mediated apoptosis and regulation of the Bcl-2 family. Expression of epithelial–mesenchymal transition (EMT) markers was regulated by TCS treatment in CT26 cells. TCS significantly inhibited the lung metastasis of CT26 cells in a mouse model. These results suggest that TCS, by inducing cell cycle arrest and apoptosis through its anti-oxidant properties, is a novel therapeutic agent that inhibits metastatic phenotypes of murine CRC cells.

## 1. Introduction

The onset of cancer is caused by environmental factors, somatic mutations, and dietary habits [1]. Colorectal cancer (CRC) is the fourth most common cause of cancer-related deaths, and CRC mortality is expected to increase in the future [2]. Unlike the epidermis, the colonic epithelium is fragile and composed of a single layer, raising the risk of cancer development [3]. Although chemotherapy, radiation, and surgery are available to treat CRC, these methods have a relapse risk and several side effects [4]. For instance, conventional cancer therapies induce DNA damage directly or indirectly to tumor cells, causing cell death; but, excluding the possibility of early tumor detection, advanced- and metastatic-cancers are resistant to therapy [5]. Consequently, researchers have become interested in finding a drug (natural compounds) that can enhance therapy response and address these issues.

**Figure 1:**
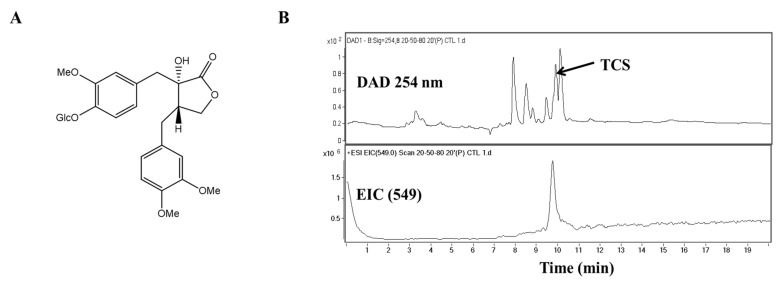
Molecular structure and chromatographic profiles at 254 nm of the tracheloside (TCS) from safflower extract. (**A**) Structure of TCS, (**B**) HPLC elution profiles and ESI mass spectra of TCS on safflower.

Cancer progression is caused by persistent oxidative stress as a result of environmental stress and leads to reactive oxidative species accumulation [6]. High levels of intracellular reactive oxidative species may cause DNA damage and initiate the epithelial–mesenchymal transition (EMT) process in cancer cells [6]. In other words, the induction of apoptosis, the inhibition of cell proliferation, and metastasis can all be achieved via anti-oxidants. Several studies are underway to develop treatments aimed at weakening DNA binding and reducing transcriptional expression. In particular, it has been suggested that anti-oxidant and anti-tumor activity are related to each other in that they both target multiple carcinogenic signaling pathways with reactive oxidative species [5]. Phenolic compounds are known to have anti-oxidant properties and have been recently reported to also display anti-tumor activity. These compounds are known as an attractive and representative resource for pharmacological and medical applications [7,8]. Thus, in this study, we confirmed the cell proliferation inhibitory effect of the phenolic compound tracheloside (TCS; Figure 1A), which is isolated from *Carthamus tinctorious* L. (safflower), in two types of human CRC cell lines and a murine CRC cell line.

Cancer cells proliferate and metastasize through uncontrolled cell growth [9]. In cancer cells, the activation of oncogenes and the deactivation of tumor suppressor genes induce cell cycle progression and block apoptotic mechanisms [9]. Malignant cancers occur through the downregulation of cell adhesion molecules and the upregulation of cell motility factors [9]. EMT arises in normal epithelial cells from various organs in response to injury and organ fibrosis and is associated with the migration and invasion capabilities of cancer cells during cancer progression [10].

The alteration of cell cycle progression causes cell malignancy and induces uncontrolled proliferation [11]. Cyclin dependent kinases (CDKs) control cell division by regulating the transition of cell cycle phases [12]. During cell cycle progression, the G1/S transition stage is the most important, and the essential complex for this step is cyclin D1/CDK4. Cyclin D1 can regulate the expression of tumor suppressor proteins by affecting gene transcription [11,13]. Cyclin D1 and CDK4 are positive regulators of cell cycle progression, whereas the CDK4 inhibitor p16 inhibits the G1/S transition in CRC cells [14,15]. Cell cycle progression is known to regulate the induction of apoptosis [16]. Bcl-2-associated X protein (Bax), a member of the Bcl-2 family, can release cytochrome c from mitochondria [17]. Cytochrome c is translocated to the apoptosome where it stimulates the cleavage of procaspase-9 to its activated form, caspase-9 [18]. Known as the initiator caspase, caspase-9 can activate caspase-3 to start apoptosis through DNA degradation and chromatin condensation [19]. Thus, the prevention of EMT and the induction of apoptosis are considered to be effective therapeutic methods for CRC.

Natural phenolic compounds present great biological activity, including anti-oxidant abilities through the stabilization of free radicals using their aromatic ring [20,21]. Moreover, recent research has reported that the safflower, by its phenolic content, revealed anti-oxidant, anti-microbial, and anti-inflammatory effects [22,23,24]. The seeds of the safflower are nutritionally similar to sunflower oil and are mainly used for cooking oil and salad dressing. In turn, they are completely non-toxic. One study has shown that safflower seeds have rich plant lignans, such as TCS, which exhibit antiestrogen activity [25]. Moreover, the pharmacological effects of safflower seeds were demonstrated to be anti-oxidant by reducing the risk of hormone-dependent cancers [26,27]. TCS is contained in safflower seeds and is similar in structure to arctigenin. Enterolactone and arctigenin are known to be effective among plant-derived components for the treatment of CRC cells [28,29]. Natural phenolic compounds are found in a variety of vegetables and fruits, which can enhance human health through regulation of DNA replication and mutation as well as the angiogenesis of cancer cells [30,31]. Previous studies have reported that TCS has anti-carcinogenic and anti-osteoporotic effects [32,33]. Little is known, however, about the mechanisms underlying the apoptosis and EMT regulation associated with the oxidative stress factors and the anti-tumor effect of TCS in CRC cells.

This study aims to discuss the inhibitory effects of TCS (Figure 1A) on CRC progression using in vitro and in vivo models and to explore the mechanisms involved in the induction of apoptosis and the regulation of EMT as anti-oxidant factors in TCS treatment.

## 2. Materials and Methods

### 2.1. Plant Materials

Tracheloside (TCS) was obtained from the seeds of *Carthamus tinctorious* L. (safflower). The isolation method for TCS (purify ≥ 99%) has been described previously [34]. Briefly, seeds were extracted and placed in methanol (MeOH) overnight. The solutions were concentrated and extracted with hexane. Then, the MeOH soluble residue of safflower extract was chromatographed on a Diaion column. The fractions containing TCS were eluted on a silica gel column.

### 2.2. Antibodies and Reagents

Antibodies against Bcl-2 and β-actin were purchased from Santa Cruz Biotechnology, Inc. (Santa Cruz, CA, USA). Antibodies against iNOS, Bcl-xL, poly-ADP ribose polymerase (PARP), cleaved-PARP, caspase-3, cleaved caspase-3, caspase-9, cleaved caspase-9, E-cadherin, N-cadherin, vimentin, snail, and twist were purchased from Cell Signaling Technology, Inc. (Danvers, MA, USA). Nrf2 and Bax antibodies were purchased from Abcam, Inc. (Cambridge, MA, USA). Annexin V assay for apoptosis analysis was performed using the FITC Annexin V apoptosis detection kit I (BD Biosciences, San Diego, CA, USA). Transwell chambers with 8 µm pore size polycarbonate membrane inserts and Matrigel were obtained from Corning (Costar Corning Inc., NY, USA) and BD Biosciences (San Diego, CA, USA), respectively.

### 2.3. High Performance Liquid Chromatography-Mass Spectrometry (HPLC-MS)

Safflower extracts and TCS isolated from the safflower seeds were analyzed by HPLC-MS. The sample was dissolved in MeOH to 1 mg/mL, and analysis was performed on an Agilent HPLC 1200 system, a photodiode array detector, an Agilent 1200 series quad pump, and an Agilent 6410 Triple Quadrupole mass spectrometer (Agilent Technologies, Waldbronn, Germany). Analysis was conducted on a TSK-gel ODS-80Ts column (Tosoh Co., Tokyo, Japan, 4.6 mm × 150 mm). Elution was applied in gradient mode with 0.1% formic acid (solvent system A) and CH_3_CN (solvent system B). The mobile phase conditions were as follows: B from 20% to 50% in 5 min, B from 50% to 80% from 5 min to 20 min, B 100% from 20 min to 25 min (0.5 mL/min flow rate). The UV wavelength was set at 254 nm. The MS spectrometer was coupled with an electrospray ionization (ESI) interface. The ESI source was operated in negative ionization mode. The system was set to a gas temperature of 300 °C, a fragmentor voltage of 150 V, a nebulizer setting of 30 psi, and a capillary voltage at ±4000 V.

### 2.4. Cell Cultures

Murine, colon 26 (CT26), human, SW480 and SW620, CRC cells, rat mast cells (RBL-2H3), and mouse macrophage (RAW264.7) cell lines were obtained from the Korean Cell Line Bank (Seoul, South Korea). CT26, RBL-2H3, and RAW264.7 cells were cultured in Dulbecco’s modified Eagle’s medium. SW480 and SW620 cells were cultured in RPMI 1640 medium. All culture media (Gibco BRL, Grand Island, NY, USA) were supplemented with 10% fetal bovine serum (Gibco BRL, Grand Island, NY, USA), 1% penicillin, and streptomycin. All cells were maintained at 37 °C in a humidified incubator containing 5% CO_2_.

### 2.5. Cell Viability Assays and Morphology Image Analysis

All cells were seeded overnight in 96-well plates. CT26 (3 × 10^3^ cells/well), SW480 (1 × 10^4^ cells/well), and SW620 (2 × 10^3^ cells/well) cells were treated with various concentrations (1, 10, and 100 μM) of TCS. RBL-2H3 cells (5 × 10^3^ cells/well) were incubated with TCS for 96 h. Cell proliferation on TCS treatment was established using water-soluble tetrazolium-1 (WST-1) reagent (Promega, Madison, WI, USA). Absorption was determined using a microplate reader (Bio-Rad, Hercules, CA, USA) and compared against the values of the control (0 μM). The percentage of viable cells in treated cells was calculated and compared to the percentage of viable cells in control cells. For morphology analysis, CT26 cells with and without 48 h of TCS exposure were photographed using an inverted light microscope at 200× magnification.

RAW264.7 cell viability was evaluated using MTT reagent (3-(4,5-dimethythiazol-2-yl)-2,5-diphenytetrazoleum bromide). RAW264.7 (3 × 10^5^ cells/well) cells were treated with TCS (1, 10, and 100 μM) for 24 h. After the 24 h incubation, MTT solution (at a final concentration of 0.5 mg/mL) was added to the medium for 4 h. The supernatant was then removed and the formazan crystals were dissolved in 500 μL of dimethyl sulfoxide. The absorbance of each well at wavelength 540 nm was then measured using the VersaMax™ microplate reader. The percentage of viable cells was then determined and compared to control cells.

### 2.6. Determination of Intracellular Reactive Oxygen Species (ROS) Accumulation

Intracellular ROS accumulation was examined in the RBL-2H3 cells (1 × 10^4^ cells/well) by seeding them into 96-well plates and pretreated with various concentrations of TCS (1, 10, and 100 μM) for 24 h. Oxidative stress was induced by adding 100 μg/mL of compound 48/80 (Sigma, St Lousis, MO, USA) for 30 min, after which the supernatant was removed and the cells were washed twice with PBS. DCFH-DA (25 μM) was mixed with 100 μL PBS and added to each well. After a 1 h incubation, relative fluorescence intensity was quantified using a fluorescence spectrophotometer (Molecular Devices, Sunnyvale, CA, USA) at an excitation wavelength of 485 nm and an emission wavelength of 535 nm.

### 2.7. Measurement of Nitric Oxide (NO) Production

RAW264.7 cells (3 × 10^5^ cells/well) were pretreated with TCS (1, 10, and 100 μM) for 3 h, followed either by no stimulation or stimulation with 100 μg/mL of lipopolysaccharide (LPS). After a 24 h incubation, the supernatants were collected to test for NO. To measure nitrite, an equal volume of Griess reagent (1% sulfanilamide and 0.1% naphtylethyenediamine dihydrochloride in 2.5% phosphoric acid) was mixed with cell culture supernatant at room temperature for 10 min. Nitrite concentration was determined and calculated using NaNO_2_ as a standard solution by measuring absorbance at wavelength 540 nm using a VersaMax™ microplate reader.

### 2.8. Annexin V Assay

Apoptotic cells were assessed using the Muse Annexin V and Dead Cell Assay kit (Millipore, Burlington, MA, USA) according to the manufacturer’s instructions. Briefly, CT26 cells were plated in 6-well plates at 1 × 10^5^ cells/well and treated with TCS (10, 25, 50, and 100 μM) for 48 h. After incubation, the cells were harvested by trypsinization and washed in phosphate-buffered saline (PBS). The cell pellets were suspended in Muse™ Annexin V and Dead Cell Reagent for 20 min at room temperature in the dark. The early and late apoptotic populations of the samples were analyzed using the Muse™ Cell Analyzer (Millipore, Billerica, MA, USA) and Muse™ analysis software. The percentage of apoptotic cells in early and late stage apoptosis was quantified.

### 2.9. Western Blotting Analysis

TCS-treated RAW264.7 cells, CT26 cells and lung tissues were lysed with a lysis buffer (iNtRon Biotech, Seoul, South Korea). The lysed samples were mixed with a 2× sample buffer and heated at 95 °C for 5 min. Proteins were separated by sodium dodecyl sulfate polyacrylamide gel electrophoresis (10% SDS-PAGE) and transferred to polyvinylidene difluoride membranes. The membranes were blocked with 5% bovine serum albumin for 1.5 h, incubated overnight with primary antibodies, and washed for 1.5 h in PBS/0.1% Tween 20. The washed membrane was incubated with horseradish peroxidase-conjugated secondary antibodies (GE Healthcare, Freiburg, Germany) for 1 h at room temperature. Prior to visualization, the membrane was washed in PBS/0.1% Tween 20 for 1.5 h. After washing, immunoreactive proteins were visualized by ImmunoCruz Western Blotting Luminol-Enhanced Chemiluminescence reagent (Santa Cruz, CA, USA) and assessed using the LAS-4000 (Fuji-film Life Science, Stamford, CT, USA). β-actin was used as a loading control. Bands of each protein were quantified using Image J (Wayne Rasband National Institutes of Health, Bethesda, Maryland, USA).

### 2.10. Cell Cycle Analysis

CT26 cells were collected for examination of cell cycle arrest after 48 h of treatment. The cell cycle phase was assessed using the Muse™ Cell Cycle Assay kit (Millipore, MA, USA) according to the manufacturer’s instructions. In brief, the cells (1 × 10^5^ cells/well) were placed in 6-well plates and treated with TCS (10–100 μM) for 48 h. The cells were collected and fixed in 70% ethanol at –20 °C overnight. Fixed cells were resuspended in cell cycle reagent in the dark for 30 min. The cells were then analyzed using a Muse cell analyzer, and the cell cycle phase distribution was determined using Muse™ analysis software (Millipore, MA, USA).

### 2.11. Real-Time Quantitative Reverse Transcription Polymerase Chain Reaction (Real-time qRT-PCR)

CT26 cells were collected from each group after TCS treatment for 48 h. To extract the total RNA, the lysates of CT26 cells were isolated using a Total RNA isolation reagent (Molecular Research Center, Inc., Cincinnati, OH, USA). The total RNA of the cells was then determined using a Nanodrop spectrophotometer (Thermo Fisher Scientific, Madison, USA). An RNA template from each group was prepared using first-strand cDNA. cDNA was synthesized using an Oligo (dT) 18 Primer and a Power cDNA synthesis kit (iNtRon Biotech, Seongnam, South Korea). Real-time RT-qPCR was performed using a SYBR (Agilent, Waldbronn, Germany) and Thermal Cycler (Life Technologies, Gaithersburg, MD, USA). The reaction mixtures were prepared according to the manufacturer’s protocol. Glyceraldehyde-3-phosphate dehydrogenase (GAPDH) was used as a housekeeping gene. The primer sequences used for real-time qRT-PCR are summarized in Table 1.

### 2.12. Wound Healing Assay

CT26 cells were seeded at a density of 1 × 10^5^ cells into 24-well plates. After incubation until 90% confluence, a scratch (linear wound) was made using a sterile yellow tip in each well, and detached cells were removed with serum-free cell culture medium. The wounded monolayer was then treated with various concentrations of TCS for 48 h. Cell migration into the wound field was photographed using an inverted microscope (magnification× 200). The wound gap closure was calculated using Image J. The wound closure rate was quantified using fold change in the measurement area divided by the initial open area according to the following formula: wound closure (fold change) = 1—(wound area at incubation time/wound area at zero time).

### 2.13. Cell Invasion Assay

A polycarbonate membrane (Costar 3422; Corning Inc., NY, USA) was used for the invasion assay. The inner part of the upper chamber was pre-coated overnight with 100 μL of Matrigel, which was diluted with PBS (0.5 mg/mL). Cells (5 × 10^5^ cells per well) were incubated with or without TCS for 48 h. CT26 cells were then suspended in serum-free medium in the upper portion with TCS for 48 h. The lower transwell chamber was filled with medium containing 10% FBS. The membranes of the upper part of the transwell chamber were washed with PBS twice and fixed with paraformaldehyde for 10 min. After washing twice with PBS, the fixed cells were permeabilized for 20 min with MeOH and stained for 15 min with Giemsa (Sigma, St Louis, MO, USA). The non-invaded cells on the inner portion of the upper chamber were wiped with a cotton swab. The invading cells of the membrane were observed under a microscope (Zeiss, Oberkochen, Germany) and counted using Image J.

### 2.14. Gelatin Zymography

Matrix metalloproteinase (MMP)-2 and MMP-9 activities were detected using a Zymogram buffer kit (Koma Biotech, Seoul, South Korea). For gelatin zymography, cells were placed in 6-well plates and cultured overnight. TCS was treated for 48 h, and the cultured medium was collected and stored at −80 °C. Samples from the supernatant were mixed with zymogram sample buffer (2×) and electrophoresed on 8% SDS-PAGE gels with 0.1% gelatin. Following electrophoresis, the gels were washed with renaturing buffer to remove SDS for 30 min at room temperature and equilibrated in developing buffer at room temperature for 30 min. Then, the gels were incubated overnight with fresh developing buffer at 37 °C. The gels were stained with Coomassie blue R-250 solution for 30 min and destained with destaining buffer. MMP activity was visualized using an imaging system under a microscope.

### 2.15. Experimental Lung Metastasis Model

Four-week-old (16–18 g body weight) female BALB/c mice (Samtaco, Osan, South Korea) were maintained in a cage under sterile conditions in a laminar air-flow room at a temperature of 22 ± 1 °C with a 12 h light and dark cycle. For the lung metastasis experiment, TCS was dissolved in 5% Kolliphor solution (D.W.: Ethanol:Kolliphor = 90:5:5). CT26 cells (5 × 10^5^ cells in 200 μL of PBS) were injected into the tail vein (intravenously) of the mice. Mice were administered with 25 and 50 mg/kg of TCS once daily. Body weight was measured on the first and last day of the experiment. Two weeks after CT26 cell inoculation, the mice were anaesthetized and sacrificed (*n* = 7 control, *n* = 6 for TCS treatment group). Lung tissues were harvested and fixed with Bouin’s solution (Sigma-Aldrich, Co., St Louis, MO, USA) to assess the anti-metastatic effect of TCS.

### 2.16. Statistical Analyses

The data are expressed as the mean ± SD of three independent experiments. All data for statistical analysis were performed using GraphPad Prism version 5.0. (GraphPad Software, Inc., La Jolla, CA, USA). All comparisons were considered using one-way analysis of variance with Tukey’s post hoc test at *p* < 0.05.

## 3. Results

### 3.1. Analysis of Tracheloside (TCS) from Carthamus tinctorious L. (Safflower) Seeds Contents

Studies are underway on using plant lignans to prevent colorectal cancer (CRC) and to avoid large side effects. TCS is a lignan glucoside (Figure 1A). When TCS and safflower extracts were analyzed by high-performance liquid chromatography-mass spectrometry (HPLC-MS), the retention time of TCS was 9.7 min. Quantitative analysis of TCS indicated that the safflower extracts contained 91.45 mg/g. The TCS mass spectrum produced a [M − H]^−^ of *m*/*z* 549 (Figure 1B). The presence of this peak confirmed the presence of TCS in the safflower extract.

### 3.2. Anti-Oxidant Effects of TCS on RBL-2H3 and RAW264.7 Cells

**Figure 2:**
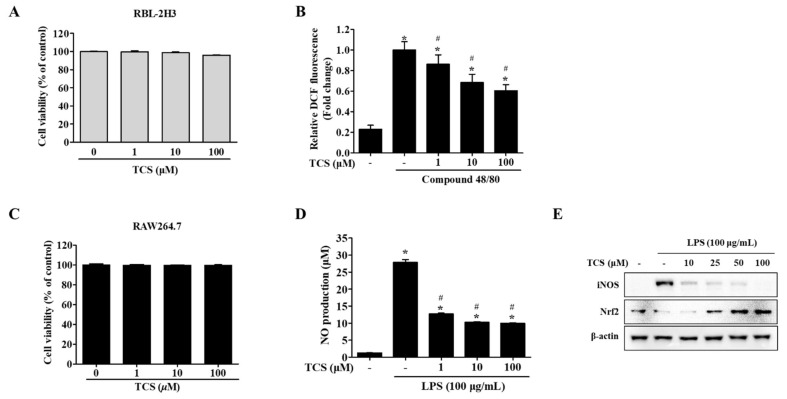
Effects of TCS on reactive oxygen species (ROS) generation (**B**) in RBL-2H3 cells after compound 48/80 stimulation and nitric oxide (NO) production (**D**), inducible nitric oxide synthase (iNOS) and nuclear factor erythroid 2-related factor (Nrf2) expression levels (**E**) in lipopolysaccharide (LPS)-stimulated RAW264.7 cells. (**A**) RBL-2H3 cells were seeded on 96-well plates and treated with TCS (1–100 μM) for 96 h to perform a water-soluble tetrazolium-1 (WST-1) assay. (**B**) RBL-2H3 cells pretreated with TCS for 24 h in compound 48/80 induced RBL-2H3 cells. The level of 2′,7′-dichlorodihydrofluorescein (DCF) fluorescence is presented as mean ± SD of three independent experiments. * *p* < 0.05 vs. control; ^#^ *p* < 0.05 vs. compound 48/80-treated cells. (**C**) RAW264.7 cells were treated with TCS for 24 h for cell viability assay. (**D**) RAW264.7 cells were pretreated with TCS for 3 h, followed by incubation with or without LPS (100 μg/mL) for 24 h. Data are expressed as mean ± SD of three independent experiments. * *p* < 0.05 vs. control; ^#^ *p* < 0.05 vs. LPS-treated cells. (**E**) RAW264.7 cells were pretreated with varying amounts of TCS (10, 25, 50, and 100 μM) for 4 h, then treated with LPS (100 μg/mL) for 24 h and subjected to Western blotting analysis with iNOS, Nrf2, and β-actin.

To evaluate the biological effects of treatment with TCS, cell viability assays using either WST-1 reagent or MTT reagent were conducted on TCS-treated mast cells (RBL-2H3 cells) and macrophage cells (RAW264.7 cells). Figure 2A,C highlight that TCS showed no cytotoxicity to the mast cell line RBL-2H3 cells after 96 h and RAW264.7 macrophage cells after 24 h.

To determine whether intracellular reactive oxygen species (ROS) were generated, RBL-2H3 cells were treated with TCS for 24 h after being stimulated with compound 48/80. Unstimulated RBL-2H3 cells (control) were found to generate a low level of ROS, while treatment with compound 48/80 induced the generation of far more ROS than were observed in control cells. Pretreatment with TCS substantially suppressed compound 48/80-stimulated ROS generation in RBL-2H3 cells (Figure 2B).

We also investigated TCS’s inhibitory effects on nitric oxide (NO) production (Figure 2D). The NO levels in the LPS treatment increased NO production to approximately 27-fold of that in the control (*p* < 0.05). In contrast, LPS-induced RAW264.7 cells treated with TCS saw a reduction in NO production in a dose-dependent manner (Figure 2D). TCS treatment in LPS-induced RAW264.7 cells also reduced inducible nitric oxide synthase (iNOS) expression (Figure 2E). As shown in Figure 2E, pretreatment with 50 and 100 μM of TCS promoted nuclear factor erythroid 2-related factor (Nrf2) expression in RAW264.7 cells.

In sum, the suppression of ROS in RBL-2H3 cells after stimulation with compound 48/80, in conjunction with the downregulation of NO levels and iNOS expression observed in LPS-induced RAW264.7 cells, indicates TCS’s anti-oxidant properties.

### 3.3. Anti-Proliferative Effects of TCS on CRC Cells

**Figure 3:**
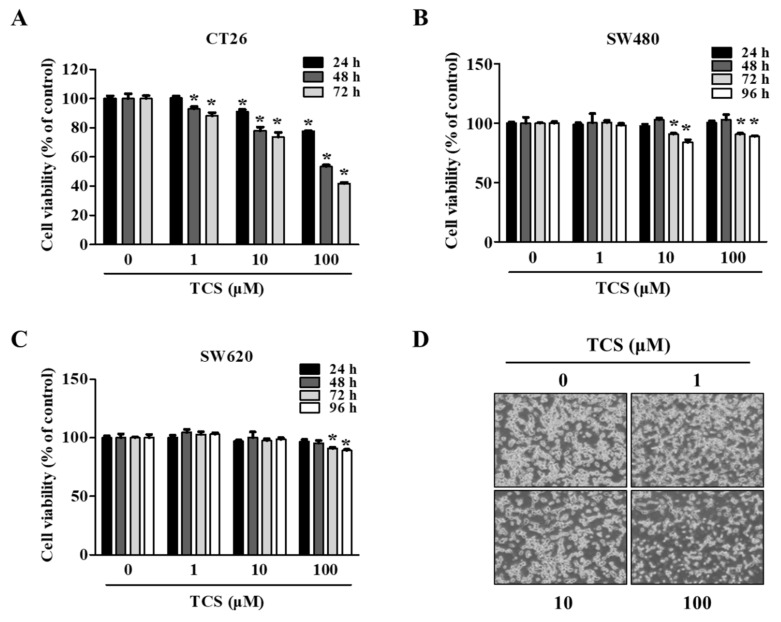
TCS decreases cell proliferation of colorectal cancer (CRC) cells. (**A**–**D**) Cell viability in CT26 (**A**), SW480 (**B**), and SW620 (**C**) on TCS treatment. Cells were seeded in 96-well plates and treated with TCS (1–100 μM). After incubation, cell viability was determined by WST-1 assay. Data are expressed as the mean ± SD of three independent experiments. * *p* < 0.05. (**D**) Morphology of TCS-treated CT26 cells for 48 h.

To evaluate the biological function of treatment with TCS, a cell viability assay using WST-1 reagent was conducted on TCS-treated CRC cells (CT26, SW480, and SW620 cells). As shown in Figure 3A, TCS (1, 10, and 100 μM) decreased CT26 viability in a dose- and time-dependent manner. Yet, the viability of the human CRC cell lines SW480 and SW620 were slightly inhibited by TCS treatment (10 and 100 μM) for 72–96 h (Figure 3B,C). TCS treatment with 100 μM significantly decreased in CT26 cells when treated for 24 h. Human CRC cells (SW480 and SW620 cells) showed a significant difference when treated for 72 h. Morphological changes in TCS-treated CT26 cells were consistently observed using microscopy (Figure 3D). These results indicate that TCS can inhibit proliferation of murine and human CRC cells.

### 3.4. TCS Induces Apoptosis and Cell Cycle Arrest in CT26 Cells

**Figure 4:**
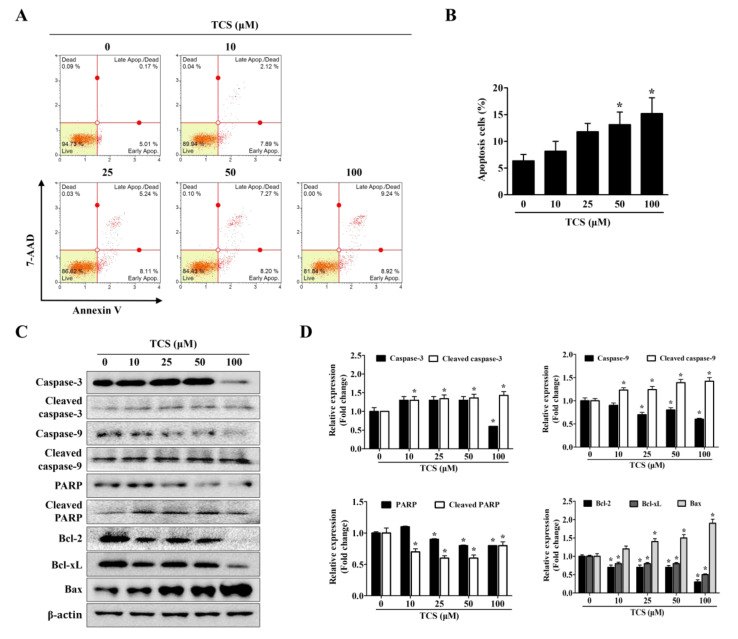
TCS induces apoptosis of CT26 cells. (**A**) Cells were incubated with TCS for 48 h and stained with Annexin V and 7-AAD. The image is representative of three independent experiments. (**B**) Percentage of apoptotic cells. Results are expressed as the mean ± SD of three independent experiments. * *p* < 0.05. (**C**) CT26 cells were treated with various concentrations of TCS (10, 25, 50, and 100 μM) for 48 h and subjected to Western blotting analysis with antibodies against caspase-3, cleaved caspase-3, caspase-9, cleaved caspase-9, poly-ADP ribose polymerase (PARP), cleaved PARP, Bcl-2, Bcl-xL, Bcl-2-associated X protein (Bax), and β-actin. (**D**) The relative expression of apoptosis-related protein levels in TCS-treated CT26 cells were analyzed using the Image J program. Data are expressed as the mean ± SD of three independent experiments. * *p* < 0.05.

To confirm whether TCS can induce cell death, apoptosis and cell cycle arrest were performed using the Muse™ analysis software (Millipore) for Annexin V assay and cell cycle analysis. In the apoptosis and cell cycle arrest analysis, CT26 cells were treated with TCS (10, 25, 50, and 100 μM) for 48 h. As shown in Figure 4A,B, TCS increased apoptosis of CT26 cells in a dose-dependent manner. For example, a 48 h exposure of CT26 cells to 10 μM of TCS led to 2.1% early apoptotic cells and 7.9% late apoptotic cells; 50 μM of TCS led to 7.3% early apoptotic cells and 8.2% late apoptotic cells; 100 μM of TCS also induced apoptosis significantly, with 9.2% early apoptosis and 8.9% late apoptosis. Apoptosis-related factors in TCS-treated CT26 cells were detected by Western blotting analysis. The expressions of cleavage of caspase-3 and caspase-9 were observed in CT26 cells exposed to TCS (10, 25, 50, and 100 μM) for 48 h. TCS also decreased the expressions of Bcl-2 and Bcl-xL, while the expression of Bax was increased in CT26 cells (Figure 4C,D). These results show that TCS significantly promoted the apoptosis of CT26 cells by regulating the expressions of anti-apoptotic proteins, Bcl-2 and Bcl-xL, and the pro-apoptotic protein, Bax.

**Figure 5:**
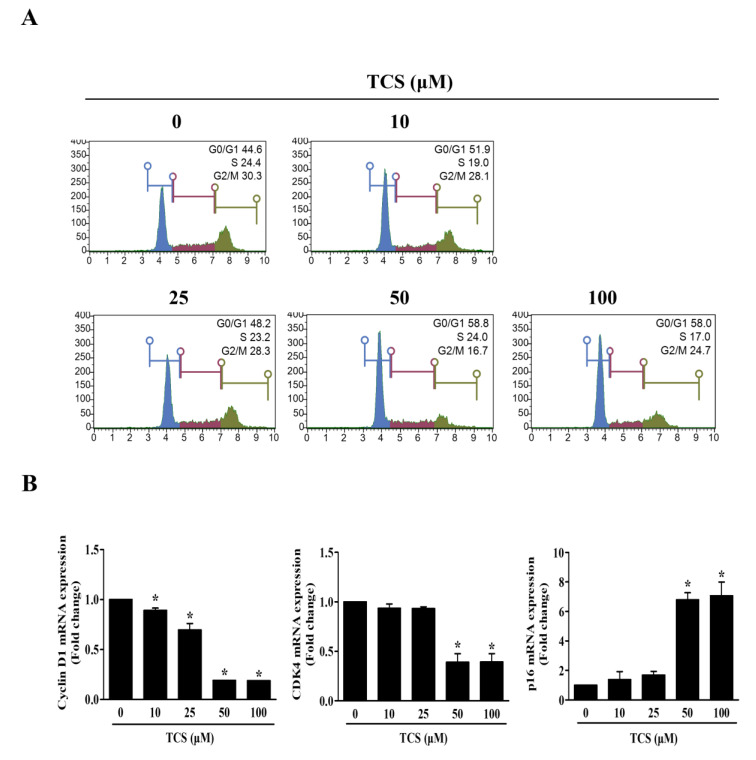
TCS causes G0/G1 phase arrest by regulating expression of cyclin D1, CDK4, and p16. (**A**) Cell cycle analysis of TCS-treated CT26 cells for 48 h. (**B**) mRNA expressions of cyclin D1, CDK4, and p16. Results are represented as the mean ± SD of three independent experiments. * *p* < 0.05.

The cell cycle also regulates cell proliferation and growth. In turn, to investigate whether TCS could induce cell cycle arrest, flow cytometry was performed to analyze the distribution of cells in each phase. Figure 5A shows that the representative cell cycle phases were changed in CT26 cells after treatment with TCS. The percentage of cells in the G0/G1 phase in CT26 cells increased at concentrations above 50 μM TCS, with 58.8%. Furthermore, the mRNA expression of cyclin D1 and CDK4 was decreased, and the mRNA expression of p16 was increased by TCS in CT26 cells (Figure 5B). These findings proved that TCS treatment induced cell cycle arrest and promoted apoptosis through the regulation of Bcl-2 family proteins and caspases in CRC cells.

### 3.5. TCS Inhibits Expression of Epithelial–Mesenchymal Transition (EMT) Markers in CT26 Cells

**Figure 6:**
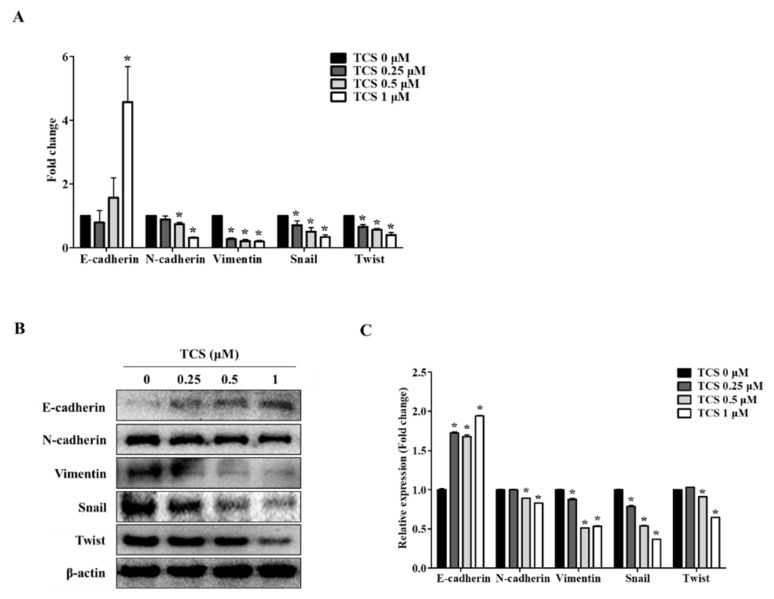
TCS regulates mRNA and protein expression of epithelial–mesenchymal transition (EMT) factors in CT26 cells. (**A**) Real-time qRT-PCR was performed to analyze the mRNA expression levels of EMT markers after treatment of TCS (0.25, 0.5, and 1 μM) on CT26 cells for 48 h. Data are the mean ± SD of three independent experiments. * *p* < 0.05. (**B**) The protein levels of EMT markers including E-cadherin, *N*-cadherin, vimentin, snail, and twist were confirmed by a Western blotting analysis. (**C**) Quantification was conducted via Image J. Data are the mean ± SD of three independent experiments. * *p* < 0.05.

We confirmed the activity of TCS in CT26 cells through EMT markers known to be associated with cell migration and invasive capabilities. Since the dose of TCS at 0.25, 0.5, and 1 μM resulted in non-toxicity in CRC cells, we investigated the effect of TCS on the metastatic phenotype of CT26 cells. As shown in Figure 6A, TCS treatment increased the expression of the epithelial marker E-cadherin and downregulated the mesenchymal markers N-cadherin, vimentin, snail, and twist. Additionally, the Western blotting analysis data was consistent with the results of real-time qRT-PCR data (Figure 6B). In addition, quantitative results for Western blotting also confirmed the inhibition of EMT-related markers (Figure 6C). These findings indicate that TCS could inhibit EMT progression in metastatic CRC cells.

### 3.6. TCS Inhibited Migration and Invasion of CT26 Cells

**Figure 7:**
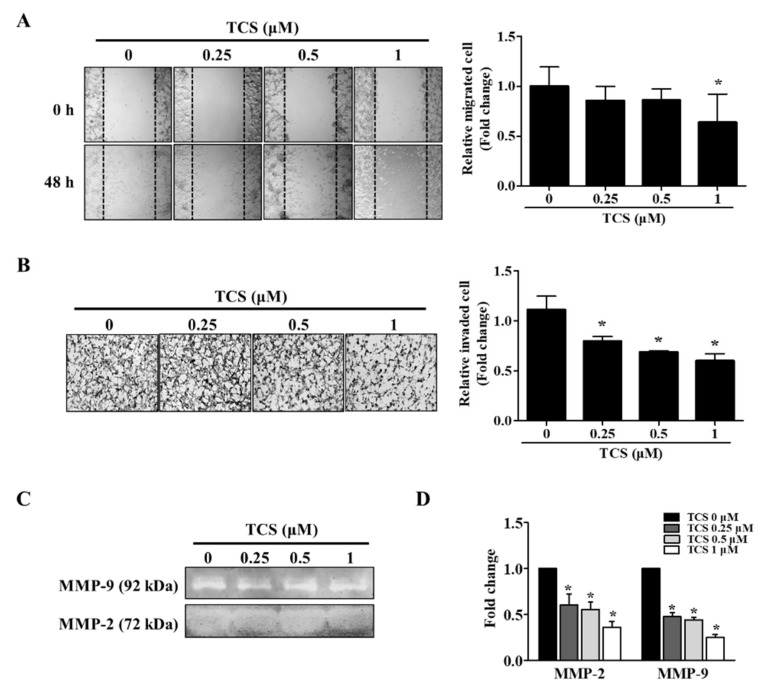
TCS reduces the migratory and invasive abilities of CT26 cells. (**A**) Wound healing assay. Images were photographed using a microscope (200× magnification). Quantitative analysis for migration was measured by Image J as average fold change in width of the wound at 48 h compared to that at 0 h. (**B**) Invasion assay. Images of the invasion assay were photographed using a microscope (400× magnification). Photographs are representative of three independent experiments. Quantitative analysis for the invasion assay counted cells that invaded the membrane by Image J and presented as the relative ratio. (**C**) Gelatin zymography. Matrix metalloproteinase (MMP)-2 and MMP-9 activity in TCS-treated CT26 cells were determined. (**D**) mRNA expression levels of MMP-2 and MMP-9 were determined after TCS treatment for 48 h. Results are expressed as the mean ± SD of three independent experiments. * *p* < 0.05.

The migratory and invasive abilities of TCS-treated cells were confirmed by wound healing assay and a cell invasion assay. As shown in Figure 7A, the non-treated cells migrated to the scratched area, whereas TCS (0.25, 0.5, and 1 μM) suppressed migration of CT26 cells in a dose-dependent manner. The inhibition rate of migration was 14.3%, 14.2%, 36.2% on 0.25 µM, 0.5 µM, and 1 µM TCS treatment in CT26 cells, respectively. Treatment with TCS decreased the invasiveness of CT26 cells compared to that in the control group, and the results were consistent with those of the migration assay (Figure 7B). In addition, TCS at 0.25, 0.5, and 1 µM doses decreased CT26 cell invasion by 28.2%, 38.1%, 46.0% respectively.

The activity and mRNA expression of MMP-2 and MMP-9 were inhibited by TCS treatment (Figure 7C,D). Taken together, these results show that TCS could suppress the metastatic phenotypes of CT26 cells, specifically their migratory and invasive activity.

### 3.7. TCS Inhibits Lung Metastasis of CT26 cells in a Mouse Model

**Figure 8:**
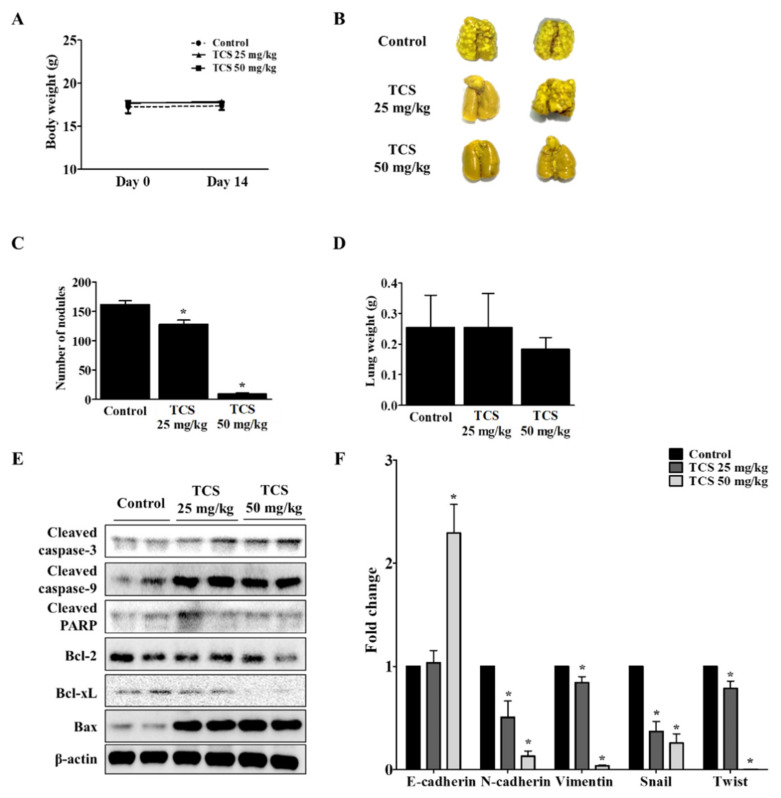
TCS inhibits colorectal lung metastasis. The mice were divided into three groups (*n* = 7 for control, *n* = 6 for TCS treatment groups) and subjected to oral administration of TCS at 25 and 50 mg/kg once a day until sacrifice. For the control group, the mice were administered the same volume of 5% Kolliphor solution. (**A**) To assess the toxicity of TCS, the body weight of the mice was monitored. (**B**) Pulmonary tumor nodule formation. (**C**) Number of tumor nodules. (**D**) Lung weight after sacrifice. (**E**) Apoptosis-related proteins in lung tissues were analyzed by Western blotting. (**F**) mRNA expression level of EMT transition markers were analyzed in lung tissues by real-time qRT-PCR. Data are expressed as the mean ± SD of three independent experiments. * *p* < 0.05.

To investigate whether TCS could suppress metastasis of CRC cells to the lung, CT26 cells were intravenously injected into the tail vein of BALB/c mice to develop an animal model of metastatic CRC. As shown in Figure 8A, mice treated with TCS (25 and 50 mg/kg) did not show significant changes in body weight, indicating the non-toxicity of TCS in mice. The number of tumor colonies and lung weight were measured to estimate the progression of metastasis in the TCS-treated group. Dose-dependent TCS treatment reduced lung weight and the number of tumor nodules in the lung (Figure 8B–D). Together, TCS decreased the expression of Bcl-2 and Bcl-xL, while the expression of cleaved caspase-3, cleaved caspase-9, cleaved PARP, and Bax increased in TCS-treated mice (Figure 8E). With respect to EMT-related markers, the mRNA expression of *E*-cadherin increased, while the expression of *N*-cadherin, vimentin, snail, and twist decreased in the TCS-treated group (Figure 8F).

## 4. Discussion

Natural phenolic compounds have beneficial effects on human health by protecting against oxidative stress-related chronic diseases [35]. Recent studies reported that phenolic extracts derived from rice bran have anti-oxidant potential by upregulating the expression of nuclear factor erythroid 2–related factor 2 (Nrf2) under oxidative stress conditions [36]. Importantly, studies on breast cancer, MCF-7, colorectal cancer (CRC) cells, SW480, HCT116, and HT29 cells have reported that reactive oxidative species play an important role in metastasis by inducing epithelial–mesenchymal transition (EMT) and mitochondrial inhibition [37,38].

In the literature, *Carthamus tinctorius* L. (safflower) has been reported to have anti-oxidant effects with phenol contents [39,40,41]. Tracheloside (TCS), a type of plant lignan, can be transformed to trachelogenin or enterolactone by microbiota in the gut when ingested [42,43]. Recent studies have shown that plant lignans such as arctigenin, trachelogenin, enterolactone, and enterodiol inhibit metastasis of CRC and breast cancer [28,29,44,45] as well as have anti-oxidant activity [46,47]. Based on the anti-tumor abilities of plant lignans, TCS is also expected to have anti-tumor activity. According to Saarinen et al. (2017), the pharmacological effect of TCS on breast cancer cells is due to its anti-estrogen effect [48]. Kim et al. (2018) have reported on how the effect of TCS improves the growth of keratinocytes [49]. The protection of keratinocytes, i.e., anti-oxidant efficacy, is associated with a reduced risk of carcinogenesis [50]. Regarding anti-oxidant activity, *Trachelospermi caulis* extract, which contains TCS or arctiin as major active components, inhibits nitric oxide (NO) and iNOS production [51]. Consistent with previous reports, the results of our tests demonstrate that TCS decreases ROS generation in RBL-2H3 cells (Figure 2B), suppresses LPS-induced NO production (Figure 2D), inhibits iNOS expression in RAW264.7 cells (Figure 2E), and promotes Nrf2 expression in LPS-induced RAW264.7 cells (Figure 2E). The authors are aware of no previous reports in the literature, however, as to whether TCS inhibits the proliferation and metastasis of CRC cells.

Cancer is caused by inappropriate cell death or complex environmental factors such as oxidative stress [52]. Initially, we assessed the anti-proliferation activity with TCS in human CRC cells and mouse CRC cells. CT26 cells are similar to the molecular features of aggressive and undifferentiated human CRC cells and are widely used in mouse tumor models [53]. Thus, CT26 cells were used to confirm metastasis inhibitory activity and were further used in mouse models. SW480 cells are derived from human primary tumors (Dukes’ stage B), and SW620 cells are derived from lymph node metastasis (Dukes’ stage C) in the same patient [54,55]. RBL-2H3 cells, basophilic leukemia cell line, exist in the colon and are known to be abundant in the cancer microenvironment. In turn, RBL-2H3 cells have been used as a model for intestinal mucosal mast cells [56,57,58].

As a result of the cell viability assay, TCS showed cell proliferation inhibition in CT26 cells without any toxicity to mast cells, which exist in the colon and infiltrate the CRC, and macrophage cells (Figure 2A,C). TCS only slightly inhibited long-term growth in human colorectal cancer cells (Figure 3B,C). According to Moura et al. (2018), however, TCS is active in human colorectal cancer cells treated with trachelogenin, the aglycone of TCS [59]. Similar to our results, arctigenin, which has a structure comparable to TCS, had a higher anti-proliferation effect in mouse-derived CRC cells than in human CRC cells [28]. In turn, some lignans are more sensitive to murine CRC cells than to human CRC cells.

When cells are damaged by toxic substances or disease states, the apoptotic response is activated [60]. Apoptosis occurs in cell populations during cell development and aging [52]. Cancer cells limit or circumvent apoptosis by removing DNA damaged sensors. Thus, induction of cell death and cell cycle arrest is important for controlling the growth of cancer cells [61]. Apoptosis is triggered by the upregulation of anti-apoptotic regulators, such as Bcl-2 and Bcl-xL, and the downregulation of pro-apoptotic proteins, such as Bax, and activation of caspases [62]. In the detection of apoptosis by the Muse™ system, a 100 μM dose of TCS treatment increased apoptotic cells about 33% versus the control group (0 μM). Cancer cells are also characterized by an imbalance in cell cycle phases [63]. When the degree of cell damage is excessive, apoptosis-related tumor suppressors can delay cell cycle progression [64]. Thus, cell cycle arrest has been proposed as a potential cancer treatment strategy. During the G1 phase, cyclin D1 and CDK4 block growth inhibitory activity and promote the cell cycle to the S phase [64]. CDKs are negatively regulated by CDK inhibitors, such as p16 [65]. p16 inhibits CDK4 activity during the G1 phase of the cell cycle [66]. CDK inhibitors also deactivate cyclin D and CDK4 during the G1/S phase [67]. The results of this experiment verify that TCS decreases the mRNA levels of cyclin D1 and CDK4, while increasing that of p16 in CT26 cells (Figure 5B). Thus, TCS is capable of inducing apoptosis and cell cycle arrest by regulating the cell cycle and apoptosis-related factors.

EMT is necessary for normal embryonic development and is also related to chronic inflammation, fibrosis cancer progression, and metastasis [67,68]. EMT features such as carcinogenesis, metastasis, and invasion are involved in CRC development [69]. Epithelial cells mediate cell adhesion and act as a barrier against external factors [67]. Expression of epithelial cell markers is reduced and mesenchymal cell markers such as snail and twist are upregulated in cancer cells during EMT [68,70,71]. Additionally, to metastasize to other organs, cancer cells invade the extracellular matrix by increasing MMP-2 and MMP-9 activity [72]. In the present study, to investigate whether TCS interferes with cancer metastasis through inhibition of EMT, we confirmed through in vitro and in vivo models using CT26 cells the effect of TCS. The results demonstrate that TCS blocked EMT of CRC cells by regulating EMT-related factors. Metastasis of CRC cells to the liver and lungs is a major cause of death. In particular, lung metastasis of CRC cells occurs more often than metastasis to other organs [73]. Having observed TCS reduce the metastatic potential of murine CRC cells, we report that this lignan glycoside is an attractive lead compound that may halt cancer progression and metastasis of CT26 cells and inhibit associated oxidative damage. Moreover, after ingestion, TCS is expected to induce apoptosis and metastasis inhibition by metabolites in the intestine.

## 5. Conclusions

As confirmed by our in vitro and in vivo experiments, tracheloside (TCS) may impede proliferation of and induce apoptosis in CT26 cells, as it inhibits associated oxidative damage. The anti-tumor effect of TCS might be partially derived from biotransformation occurring through the gut microbiota. This study confirmed, for the first time, that TCS has anti-proliferative and anti-metastatic effects on colorectal cancer (CRC) cells. TCS induced apoptosis of CT26 cells through caspase activation and regulation of Bcl-2 family proteins. In addition, downregulation of cyclin D1/CDK4 and upregulation of p16 lead to G0/G1 phase arrest by TCS treatment. TCS also suppressed metastatic phenotypes such as epithelial–mesenchymal transition (EMT), as well as the migration and invasion of CT26 cells. Thus, TCS reduced lung metastasis of CT26 cells by inducing apoptosis of cancer cells and controlling EMT-related factors in a mouse model. Our results suggest that TCS, through its anti-oxidative properties, may be an effective therapeutic agent against murine CRC.

## Figures and Tables

**Table 1:** Sequence of real-time RT-qPCR primers.

GenBank^®^ Accession Number	Gene	Primer Sequence (5′-3′)	
		Forward	Reverse
NM_001379248	Cyclin D1	TAGGCCCTCAGCCTCACT	CCACCCCTGGGATAAAGC
NM_001355005	CDK4	AGAGCTCTTAGCCGAGCG	TTCAGCCACGGGTTCATA
NM_001040654	p16	AATCTCCGCGAGGAAAGC	GTCTGCAGCGGACTCCAT
NM_013599	MMP-9	AGACCAAGGGTACAGCCTGTTC	GGCACGCTGGAATGATCTAAG
NM_008610	MMP-2	CCCCATGAAGCCTTGTTTACC	TTGTAGGAGGTGCCCTGGAA
NC_000074	E-cadherin	AATGGCGGCAATGCAATCCCAAGA	TGCCACAGACCGATTGTGGAGATA
BC133731	N-cadherin	TGGAGAACCCCATTGACATT	TGATCCCTCAGGAACTGTCC
NM_001287023	Vimentin	CGGAAAGTGGAATCCTTGCA	CACATCGATCTGGACATGCTG
AY667392	Snail	TCCAAACCCACTCGGATGTGAAGA	TTGGTGCTTGTGGAGCAAGGACAT
KP013755	Twist	AGCTACGCCTTCTTCGTCT	TCCTTCTCTGGAAACAATGACA
GU214026	GAPDH	CGTATTGGGCGCCTGGTCAC	ATGATGACCCTTTTGGCTCC

## Data Availability

The data are available from the corresponding author upon request.

## Abbreviations

7-AAD	7-aminoactinomycin D
CT26	Colon 26
CRC	Colorectal Cancer
CDKs	Cyclin-Dependent Kinases
DCF	2′,7′-Dichlorodihydrofluorescein
EMT	Epithelial–Mesenchymal Transition
ESI	Electrospray Ionization
GAPDH	Glyceraldehyde-3-Phosphate Dehydrogenase
HPLC-MS	High Performance Liquid Chromatography-Mass Spectrometry
iNOS	Inducible Nitric Oxide Synthase
LPS	Lipopolysaccharide
MeOH	Methanol
MMP	Matrix Metalloproteinase
NO	Nitric Oxide
Nrf2	Nuclear Factor Erythroid 2-Related Factor 2
PARP	Poly-ADP Ribose Polymerase
PBS	Phosphate-Buffered Saline
Real-time qRT-PCR	Real-Time Quantitative Reverse Transcription Polymerase Chain Reaction
SDS-PAGE	Sodium Dodecyl Sulfate Polyacrylamide Gel Electrophoresis
TCS	Tracheloside
WST-1	Water-Soluble Tetrazolium-1
